# The Gastrointestinal Frontier: IgA and Viruses

**DOI:** 10.3389/fimmu.2013.00402

**Published:** 2013-11-28

**Authors:** Sarah E. Blutt, Margaret E. Conner

**Affiliations:** ^1^Department of Molecular Virology and Microbiology, Baylor College of Medicine, Houston, TX, USA

**Keywords:** IgA, rotavirus, calicivirus, norovirus, adenovirus, astrovirus, small intestine, gastrointestinal virus

## Abstract

Viral gastroenteritis is one of the leading causes of diseases that kill ~2.2 million people worldwide each year. IgA is one of the major immune effector products present in the gastrointestinal tract yet its importance in protection against gastrointestinal viral infections has been difficult to prove. In part this has been due to a lack of small and large animal models in which pathogenesis of and immunity to gastrointestinal viral infections is similar to that in humans. Much of what we have learned about the role of IgA in the intestinal immune response has been obtained from experimental animal models of rotavirus infection. Rotavirus-specific intestinal IgA appears to be one of the principle effectors of long term protection against rotavirus infection. Thus, there has been a focus on understanding the immunological pathways through which this virus-specific IgA is induced during infection. In addition, the experimental animal models of rotavirus infection provide excellent systems in which new areas of research on viral-specific intestinal IgA including the long term maintenance of viral-specific IgA.

## Introduction

Gastrointestinal infections kill about 2.2 million people each year worldwide (1). In the United States, between 60 and 70 million are affected annually with gastrointestinal diseases (2) and viral gastroenteritis ranks among the top 15 principal discharge diagnoses from hospital admissions (3). Viral infections of the gastrointestinal tract are divided into two broad categories based on whether infection results in disease there (enteropathogenic) or elsewhere (non-enteropathogenic). Classical enteropathogenic viruses actually infect cells that comprise the gastrointestinal system resulting in gastrointestinal disease symptoms such as vomiting, diarrhea, malabsorption, and pain. The majority of viral gastrointestinal illnesses are caused by rotavirus, norovirus, adenovirus, and astrovirus. By contrast, although non-enteropathogenic viruses enter the body via the gastrointestinal tract, they cause mild to no gastrointestinal disease because they are distributed systemically and cause disease in other organ systems. Examples of important human non-enteropathogenic viruses include polio virus, coxsackievirus, echovirus, and hepatitis A virus. Although not a perfect fit in either category, HIV can enter through the lower gastrointestinal tract and can be associated with mild gastrointestinal disease. HIV infects cells of the immune system both in the gastrointestinal tract and systemically and thus its most severe effects are on the immune system. Both enteropathogenic and non-enteropathogenic gastrointestinal viruses induce IgA that functions in protective immunity. This review will focus on enteropathogenic gastrointestinal virus infections highlighting rotavirus, since much has been learned from the experimental animal models. The role that gastrointestinal IgA plays in protective immunity and the mechanisms through which intestinal IgA is induced will be discussed. Emerging areas in IgA research during viral gastrointestinal infections will be explored.

## IgA and IgA Deficiency in the Gastrointestinal Tract

IgA is one of the main effector molecules produced by initiation of immune responses in the gastrointestinal tract. IgA is predominant in the intestinal lumen and it is synthesized in quantities that far exceed any of the other antibodies (4). Despite high levels of IgA in the gastrointestinal tract, its importance in intestinal immunity to pathogens has been difficult to prove. IgA clearly functions in binding to antigens, toxins, foreign proteins, and microorganisms to inhibit penetration of the intestinal epithelium (5–11). Intestinal IgA is also critical for regulation of commensal bacterial populations (12) and in its absence these populations expand, eventually escaping the gastrointestinal tract, resulting in both local and systemic activation of the immune system (12–14). By containing and controlling the microorganism population, IgA prevents their access to the intestinal immune system and thus functions to reduce local inflammation induced by endogenous bacteria (15).

IgA deficiency is the most common primary immunodeficiency although incidence depends on genetic background (16). IgA deficiency ranges from 1:223 to 1:1000 in community studies and from 1:400 to 1:3000 in healthy blood donors (17–19). Selective IgA deficiency is defined by serum levels of IgA below 0.05 g/L (19, 20). Low levels of IgA have been associated with a range of clinical manifestations including increased incidence of gastrointestinal diseases such as giardiasis, malabsorption, lactose intolerance, celiac disease, ulcerative colitis, lymphoid hyperplasia, and malignant proliferation (21–24). Patients with IgA deficiency suffer from an increased incidence of gastrointestinal infections and multiple bouts of diarrhea compared to IgA normal individuals (25–27). Despite these general statements, there are no well controlled studies that address the question of whether or not IgA deficiency predisposes individuals to increased susceptibility to and recurrence of gastrointestinal viral infections. In fact, it is estimated that 85–90% of IgA-deficient individuals are asymptomatic (25). One explanation might be that individuals with low levels of serum IgA may actually have sufficient secretory IgA at their mucosal surfaces to remain asymptomatic (28, 29). Another might be that other antibody isotypes, in particular IgM, via transport to the mucosal surface, compensates for the loss of IgA (30–32).

## IgA and Protective Immunity Against Gastrointestinal Viral Infections

Since IgA is produced in large quantities at mucosal surfaces including the gastrointestinal tract, it has long been presumed that IgA is a critical factor in protection of these surfaces against viral infections. Many studies in humans correlate increases in viral-specific IgA levels at the mucosal surface with either the cessation of virus excretion or protection against infection and disease (33–37). With the lack of an overt clinical profile in IgA-deficient humans, it has been difficult to discern the importance of IgA in the immune response to gastrointestinal viruses. Adding to this difficulty are the relatively few animal models of enteropathogenic or non-enteropathogenic gastrointestinal virus infections in which the pathogenesis and immune response, including IgA induction to the virus, faithfully models infection in humans. There are several reasons for the lack of robust animal models. Several of the common enteropathogenic and non-enteropathogenic viruses only replicate in humans or primates, limiting studies that can be performed to determine IgA importance (38). Other viruses infect non-primate animals but the pathogenesis is dramatically different from infection and disease in humans (38), leading to questions regarding the relevance of conclusions drawn from these models to human health.

Non-enteropathogenic viruses invade the body by either breeching or crossing the epithelium of the gastrointestinal tract (39–43). Although present in the gastrointestinal tract, in most cases these viruses do not infect a significant number of cells but once they have crossed the gastrointestinal tract barrier, disseminate systemically to access secondary target sites of viral replication (42). Because these viruses are able to spread systemically, IgG usually plays a significant role in protective immunity (44–50). However, there is evidence linking mucosal IgA to protective immunity for some of these viruses (Table 1).

**Table 1:** **Role of IgA in protective from non-enteropathogenic and pathogenic intestinal viral infections**.

Virus	IgA			
	Induced by infection (natural/exp)	Correlate of protection		Required for protection
		Humans	Animals	
**NON-ENTEROPATHOGENIC**				
Poliovirus	Y	Y	?	?
Coxsackievirus	Y	?	?	?
Echovirus	Y	?	?	?
Hepatitis A	Y	?	?	?
Reovirus	Y	?	Y	Y
HIV	Y	Y/N	Y/N	?
**ENTEROPATHOGENIC**				
Rotavirus	Y	Y	Y	Y
Calicivirus	Y	Y/N	?	?
Adenovirus	Y	?	?	?
Astrovirus	Y	?	?	?

*Y, yes; N, no; ?, unknown*.

Poliovirus, a good example of a non-enteropathogenic gastrointestinal virus, induces a secretory IgA response that appears to neutralize the virus and is associated with decreases in virus shedding in stool (51–55). Mucosal IgA correlates with protection against polio infection (56). An intestinal IgA response is also induced with the live replicating oral polio vaccine (OPV) and it is surmised that OPV prevents infection through IgA-mediated viral neutralization in the intestine. Live poliovirus vaccine appears to induce long-lived memory immune responses as elderly people who received this vaccine and still had detectable serum and salivary IgA were resistant to reinfection (56). In addition, IPV vaccination of individuals that were 20–44 years of age and had previously been vaccinated with OPV induced IgA^+^ α4β7^+^ antibody secreting cell by 7 days post vaccine but not in individuals of similar age previously vaccinated with IPV, which does not induce intestinal IgA responses. These data are consistent with the induction of memory IgA responses in the intestine. Therefore, whether IgA memory in the intestine to enteric viral pathogens undergoes the continuous adaptation observed with a commensal organism (57) is still an open question. For other non-enteropathogenic viruses, such as hepatitis A, coxsackievirus, and echovirus, much less is known about the relative importance of mucosal IgA in protective immunity (Table 1).

Reovirus, although not causing significant disease, is often used as a model system of non-enteropathogenic infection in mice. In reovirus infection, following binding and transport across M cells in the Peyer’s patches, the virus is distributed systemically where it can cause disease (58). It also infects the adjacent intestinal epithelium at the basolateral membrane (59) but is not an important cause of gastrointestinal disease in humans Reovirus infection induces intestinal IgA production and IgA protects against infection when administered orally at the same time as the virus or when secreted from subcutaneous tumors (9, 60). Mice lacking expression of IgA are susceptible to reinfection with reovirus (11) indicating that IgA is an essential component of immune protection. Whether the reovirus model faithfully predicts the role of IgA in immunity to other non-enteropathogenic viruses awaits definitive proof. Protective immunity against most non-enteropathogenic infections has focused on systemic immune responses or the immune response at the site of disease rather than on the mucosal IgA response. The lack of adequate animal models has severely limited insights into the relative importance of mucosal IgA in protective immunity to these viruses.

Enteropathogenic gastrointestinal viral infections are the major cause of diarrhea and vomiting disease in humans worldwide and most induce IgA within the first week after viral exposure. Astroviruses and adenovirus are important causes of acute gastroenteritis primarily in infants and young children as well as in the elderly and immunocompromised patients (61–68). There is limited evidence that protection from both adenovirus and astrovirus infections correlates with mucosal virus-specific IgA in humans (34, 69, 70). These viruses lack small animal models, in which pathogenesis and immunity is similar to that observed in humans. The number of cases and disease severity of gastroenteritis caused by these two viruses is far less than that caused by calicivirus (71–74).

Two genera of caliciviruses, norovirus and sapovirus cause infectious gastroenteritis (75). Sapoviruses cause gastroenteritis in young children and in long term health care settings but the number of cases is far less than noroviruses (75). Norovirus is becoming the predominant cause of viral diarrhea in all age groups worldwide (76) and is the causative agent of >96% of all outbreaks of non-bacterial gastroenteritis (77). Epidemiological data gathered from human studies suggests a link between mucosal IgA induced either by infection or by non-replicating vaccines and short term protective immunity from norovirus infection (78–80). Elucidation of the role of the gastrointestinal IgA response to norovirus has been limited by the absence of animal models in which human noroviruses replicate or that mimic the course of gastrointestinal infection and disease in humans. Several non-human primate models have been developed with limited success in advancing knowledge of clinical infection and illness arising from norovirus infection (81–84). More has been gleaned from the gnotobiotic pig and calf models which exhibit diarrhea, virus shedding in feces, seroconversion, and immunocytopathic changes in the intestine (85–87). In norovirus infected gnotobiotic pigs, anti-norovirus IgA is detected as early as 6 days following virus exposure and diarrhea and severity moderately correlated with convalescent phase intestinal antibody IgA titers (87). Similarly, both norovirus-specific IgA and IgA secreting cells were present 28 days following norovirus exposure in gnotobiotic calves (86). Unlike norovirus infection in gnotobiotic piglet and calf, murine norovirus infection differs substantially from human norovirus pathogenesis, clinical manifestations, host receptors, and infected cell types (88) but a requirement for B cells and antibody in timely virus clearance and vaccine-induced protection is implicated in the mouse (89, 90). The limitations of these animal model systems, such as the lack of intestinal microbiota and differences in pathogenesis, have precluded elucidation of whether mucosal IgA is likley protective in human infection and disease.

Like norovirus, rotavirus is a major cause of gastroenteritis especially in pediatric populations where the disease is most severe. Rotavirus accounted for nearly half a million deaths in children younger than 5 years old worldwide each year prior to introduction of vaccines (91). However, unlike norovirus, mucosal rotavirus-specific IgA strongly correlates with less severe disease and prevention of rotavirus infection in humans (37, 92–95). The differences in pathogenesis between these two viruses and the lack of good reagents and model systems in which to advance our limited understanding of the pathogenesis and immune response to norovirus infection potentially explain these correlative differences. Rotavirus-specific IgA has been shown to neutralize the virus as well as mediate heterotypic protection (96). Vaccine development strategies primarily focused on utilizing live attenuated strains that replicate in the intestine and have been successful likely because of the induction of mucosal IgA responses. Unlike norovirus, there are excellent animal models of rotavirus infection and disease that range from horses to rodents that mimic human disease. From these models (discussed in more detail in the coming sections below), virus induced IgA has been shown to play an important role in clearance of infection and protection from reinfection.

## Mechanisms of IgA Induction

Protective IgA responses to gastrointestinal viruses are thought to be comprised of high affinity antibodies that recognize and neutralize the viruses. High affinity IgA producing cells arise from the actions of helper T cells, within the context of the germinal center environment in the gastrointestinal inductive sites, Peyer’s patches, isolated lymphoid follicles, and mesenteric lymph nodes (97). These helper T cells signal B cells using such molecules as TGFβ and CD40, to induce class switch recombination and somatic hypermutation resulting in the production of high affinity IgA (98) that is thought to function to neutralize the intestinal virus. Once signaled to become a high affinity IgA^+^ B cell, the cell leaves the inductive site germinal center and circulates back to the intestinal lamina propria based on cell surface expression of markers, such as α4β7 (97, 99–101). This process takes at least 7–10 days following initial virus infection in the gastrointestinal tract.

In contrast to the production of high affinity IgA that results from interactions between T and B cells in the germinal center environment, unmutated low affinity IgA can be synthesized very rapidly in the gastrointestinal tract in a T cell independent fashion (102, 103). This low affinity antibody primarily functions to limit penetration of commensal microbes through epithelial cells (104) and most believe that it does not play an important role in limiting pathogens, including gastrointestinal viruses. However, virus-specific intestinal IgA, that is presumably high affinity, develops rapidly and many acute viral infections are resolved prior to the time frame required for generation of germinal center high affinity IgA antibody. Mice that have defects in germinal center formation develop specific intestinal IgA responses, including to viruses (105–107), providing further evidence that germinal center reaction might not be necessary for clearance of infection and production of virus-specific antibody. Therefore, an alternate possibility is that IgA generated through T cell independent pathways develops sufficient affinity to limit viral replication. Rapid T cell-independent virus-specific antibody responses are generated during many acute virus infections, including VSV, influenza, and polio (108–116). These antibodies mediate virus clearance and limit replication and dissemination prior to generation of T cell-dependent IgA (117). Virus-specific IgA can be induced in the absence of CD4^+^ T cells (110, 118, 119). Mice lacking expression of MHC II (120), CD40 (121), or CD40L (122) can exhibit antibody class-switching and it is thought that molecules such as BAFF and APRIL produced by dendritic and epithelial cells drive class switch recombination and somatic hypermutation in B cells independently of germinal centers (123–127). Emerging evidence implicates a greater role for T cell independent non-germinal center generated IgA in pathogen-specific responses in the intestine.

## Rotavirus: A Model System to Study Intestinal IgA Induction

Rotavirus is a highly infectious double-stranded RNA virus that replicates in epithelial cells of the small intestine (128–131). Virus is excreted in the stool and is transmitted from infected individuals by the fecal oral route. Infection, measured by excretion of the virus in stool, lasts on average 3–8 days and is manifested by fever, emesis, and diarrhea. Disease is most severe in the young, the elderly, and the immunocompromised. Rotavirus-specific intestinal IgA is one of the principle effectors of long term immunity based on correlative studies (37, 92–95). Rotavirus is one of the few gastrointestinal viral infections in which the pathogenesis and immune response in experimental animal models closely mimics that of humans. Virtually all naïve individuals and animals are susceptible to rotavirus infection but rotaviruses exhibit some species specificity. All of the animal models of rotavirus infection and disease (horse, cow, sheep, gnotobiotic piglet, rat, and mouse) exhibit the same primary tropism of virus to the small intestinal epithelial cells, excretion of the virus in the stool, kinetics of infection, most severe disease in the young, and induction of rotavirus-specific intestinal IgA that correlates with clearance of infection and protective immunity (129–149). Both humans and animals exhibit widespread systemic distribution of the virus (141, 150). The similarity of pathogenic features of disease and immunity between rotavirus infection in humans and across all animal models is nearly unique among gastrointestinal viral and bacterial infections making the rotavirus experimental animal models excellent systems for understanding human pathogenesis and immunity.

## Animal Models

The gnotobiotic piglet and the mouse model are the principle models of experimental rotavirus infection. Gnotobiotic piglets exhibit diarrheal disease after infection with multiple porcine and at least one human rotavirus strains and disease severity diminishes with age (136). Protection against human rotavirus infection correlates with both serum and intestinal rotavirus-specific IgA levels and antibody secreting cells in this model (136). Mice of all ages, irrespective of genetic background, are susceptible to murine rotavirus infection (e.g., EC_wt_, EDIM, and McN) but have limited susceptibility to non-murine strains of rotavirus (132, 151–153). Rotavirus-associated diarrheal disease in mice is age restricted and is observed only up to 2 weeks of age (151). Following inoculation of adult mice with murine strains, rotavirus is detectable in stool by 24–48 h and systemically in the blood between 48 and 72 h (154). Mice resolve infection between 5 and 7 days after viral inoculation concurrent with the detection of stool IgA (151). Rotavirus-specific IgA is the predominant immunoglobulin response in the intestine and IgA titers persist long term. Mice are completely protected from reinfection for the lifetime of the mouse (155).

## Passive Protection

Passive protection from rotavirus has been demonstrated in many animal model systems and early studies indicated that it is primarily mediated by presence of antibody in the intestine not in the circulation (156–158). In mice, IgA was shown to be more potent than IgG in protecting pups from rotavirus disease but protection was observed with both isotypes (156). Passive protection is mediated by neutralizing antibody to two rotavirus neutralization antigens VP4 and VP7 (159) but an IgA monoclonal antibody to VP6 administered by backpack also protects through intracellular neutralization (160–162). Passive protection of infants and toddlers from nosocomial infection or during an outbreak in an orphanage has been assessed in several small studies in which children were administered either human gammaglobulin or bovine colostrum from hyperimmunized cows (163–166). Significant protection was observed in some but not all studies. Whether protection was mediated by rotavirus-specific IgA or IgG is not known. A role for IgA in passive protection of children from rotavirus has been suggested based on breast feeding studies but not all studies have supported the protective effects of breast feeding against rotavirus (167–171).

## Protection Induced by Vaccines

During the development of both non-replicating and replicating rotavirus vaccine candidates, IgA has been explored as major correlate of protective immunity induced by rotavirus vaccines (139, 153, 172–185). However, demonstration of a conclusive role for vaccine-induced IgA in protective immunity against infection and disease in children has remained elusive (186, 187). In the last 7 years, Rotarix (GSK Biologicals) and RotaTeq (Merck), two live oral rotavirus vaccines were licensed for use and have been show to prevent severe disease and death in children (188–192). Lower levels of vaccine-induced serum IgA titers correlate with higher child mortality (193). In addition, vaccine efficacy and duration of protection could be predicted by serum IgA titers. What is lacking in these analyses is whether the levels of serum IgA induced by the rotavirus vaccines can accurately predict protection from infection. One caveat to determining the role of vaccine-induced IgA in protective immunity may be that in humans, there are differences between serum IgA and IgA present in the intestine, the most significant being that serum IgA is a different isoform than the IgA present at mucosal surfaces. Further studies are necessary to identify whether vaccine-induced serum or mucosal IgA are true effectors of rotavirus protective immunity.

## IgA and Rotavirus

The conclusion that gastrointestinal IgA is critical for resolution of rotavirus infection and in protection from reinfection is drawn mainly from studies in gene knockout mice. Mice lacking B cells exhibit significant delays in clearance of a rotavirus infection (194) and fail to establish protective immunity against a second infection (140). These deficits contrast to the limited to no defects in clearance or protection in mice lacking T cells (118, 195). High levels of protection against rotavirus infection are induced in mice lacking T cells, T cell knockout animals produce ~60% of wild type rotavirus-specific IgA (118). T cell independent antibody plays a role in resolution of infection (196). Mice without IgA exhibit similar delays in viral clearance and in the development of protective immunity (197), leading to the conclusion that IgA is critical. Further conclusions about the importance of mucosal IgA to rotavirus immunity come from studies in mice lacking the ability to transport IgA and IgM from the intestinal lamina propria to the lumen due to the absence of J-chain expression, a protein required for transport. J-chain knockout mice exhibit an almost identical delay in clearance and absence of protective immunity to that reported in both B cell and IgA knockout mice (179). Together these studies indicate that IgA in the intestinal lumen is a key component in the immune response to rotavirus.

**Figure 1:**
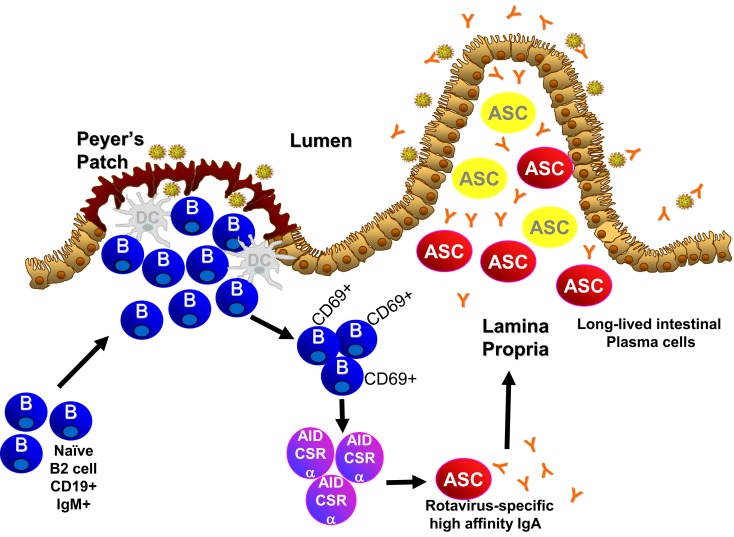
**Novel pathway of IgA induction and longevity in the intestine**.

Emerging work has focused on the type and origin of B cells that are required for the IgA response to rotavirus. Adoptive transfer studies where α4β7^−^and α4β7^+^ B cells were transferred into mice chronically infected with rotavirus demonstrate that α4β7 expression, which is expressed on B cells that circulate to the intestinal lamina propria, plays an important role in clearance of an ongoing rotavirus infection (198). In addition to α4β7, recruitment of B cells to the intestine also depends on expression of CCR9 and CCR10, receptors for ligands CCL25 and CCL28 which are exclusively expressed in the gastrointestinal tract (199). Circulating B cells that ultimately reach the intestine can originate from several sources including the peritoneal cavity (B1 cells), the Peyer’s patches (naïve B cells), and the bone marrow (memory B cells). Data in the gnotobiotic piglet model suggests that bone marrow B cells do not play a significant role in rotavirus immunity (200). Although B1 cells are a major source of intestinal antibody, B1 cells are not critical to the IgA response to rotavirus (201). B cells in the Peyer’s patches are currently being studied and hold great potential for understanding fundamentals of induction of the IgA response to rotavirus. Peyer’s patches are inductive sites for mucosal IgA responses in the small intestine (202). Rotavirus induces specific IgA antibody in the PP and this precedes appearance of IgA in the lamina propria (101), suggestive that rotavirus-specific antibodies originate in the PP and not the lamina propria. This is supported by studies in mice that lack expression of the TNF family member LTα that do not develop Peyer’s patches (203) and these mice are unable to clear rotavirus infection or produce stool rotavirus-specific IgA following virus exposure (204). This is similar to the response seen in B cell, IgA, and J-chain knockout animals (140, 179, 194, 197). Therefore, Peyer’s patch B cells appear to be critical for intestinal rotavirus-specific IgA. Analysis of the Peyer’s patches of mice within 24–48 h after infection indicates there are large increases in activated B cells (205), which may be driven by type I interferons secreted by dendritic cells (206). This activation is followed by an increase in local production of rotavirus-specific antibody (205). Similar levels of Peyer’s patch B cell activation and early antibody production occur in the absence of T cells (205), indicating that the early Peyer’s patch B cell response to rotavirus is T cell-independent with minimal T cell activation and inflammatory response induced during infection (205, 207). Since T cells are a mainstay of the germinal center environment, T cell independence suggests the lack of germinal center involvement in the rotavirus induced B cell activation and IgA induction. Mice lacking the classical germinal center molecules CD40 and CD40L produce wild type levels of intestinal rotavirus-specific IgA (107). Indeed, the first rotavirus-specific B cells detected in the Peyer’s patch early after infection are extra-follicular B cells (101). Therefore, rotavirus activates Peyer’s patch B cells and induces IgA through non-classical T cell independent extra-follicular pathways (Figure 1).

Although experimental models of rotavirus infection have been used to characterize the B cell and IgA response to rotavirus, there is still more to learn from these models about the role of IgA in gastrointestinal virus infections. Little is known about signals that direct PP B cells toward extra-follicular growth instead of germinal center formation during rotavirus infection in the mouse. Classically, TGFβ and signaling through CD40 modulate the switch to IgA in the germinal center with the help of cytokines such as IL-10, L-6, IL-4, and IL-2 produced by T cells (103). Consistent with the hypothesis that germinal centers are not playing a large role, IL-6 is not required for rotavirus-specific IgA production or virus immunity (208). It remains to be discerned whether any of the other cytokines linked to IgA induction are critical to or affect mucosal IgA levels to rotavirus. The evidence that rotavirus induces extra-follicular B cells that do not rely on T cell help raises the possibility that other factors are necessary to direct the B cell response. BAFF and APRIL are candidates as well as yet unidentified factors (125, 209). Rotavirus infection in mice lacking BAFF and APRIL expression should determine whether these molecules are critical for the intestinal IgA response to rotavirus. Interestingly, absence of CCR6 results in a 70–80% decrease in rotavirus-specific IgA levels compared to wild type mice (210). CCR6 is critical for localization of dendritic cells to the subepithelial dome of the Peyer’s patch (210) raising the question as whether Peyer’s patch dendritic cells regulate induction of B cell activation and IgA responses during rotavirus infection. Rotavirus infection activates dendritic cells in the Peyer’s patches prior to and concurrent with the activation of the B cells (211) and activation of B cells is dependent on dendritic cells (206). The Peyer’s patch activated dendritic cells produced IL-10, IL-12/23, and TNFα and upregulate expression of INFα and INFβ but B cell activation appears to be dependent on type I IFN (206, 211). The dendritic cell derived signaling contributed to the IgA response to rotavirus (206).

## Use of the Rotavirus Animal Models to Open New Areas of Research into Viral-Specific IgA

Because rotavirus induces a profound intestinal IgA response, the rotavirus model systems are valuable tools in which the plasticity of the IgA repertoire can be explored. Almost nothing is known about how the diversity and specificity of IgA in the gastrointestinal tract is shaped or altered during viral infections. There has been some indication, using methods that involve expansion of single rotavirus-specific circulating B cells, that there is low number of somatic hypermutation in circulating rotavirus-specific IgA^+^ B cells isolated from infants and adults that have previously experienced a rotavirus infection and that VH1–46 is the immunodominant gene segment, except in CD5^+^ B cells in young children (212–214). The availability of more rapid and broad sequencing approaches provides a new methodology to probe and understand the diversity and composition of the intestinal IgA repertoire. Using high throughput sequencing, the IgA producing plasma cell pool in the small intestine was recently demonstrated to contain two subfractions: frequent oligoclonal plasma cells that have low diversity and are present in high numbers and polyclonal plasma cells that have high diversity but are present in low amounts (215). Analysis of over one million Vh sequences extracted from plasma cells in the proximal, middle, and distal portions of the small intestine revealed that there are both highly expanded IgA secreting plasma cells (clonally related) as well as low frequency clones (clonally unrelated). The authors concluded that there is more diversity in the IgA repertoire than has previously been demonstrated. It remains to be determined how a highly pathogenic viral infection that induces a substantial IgA response alters the plasma cell composition. In both animals and humans, rotavirus-specific IgA, once induced, can persist for long periods of time (136, 155, 216–220). This persistence suggests that rotavirus infection makes a permanent alteration to the IgA repertoire. Next-generation sequencing is a powerful new tool which has the potential to reveal pivotal insights as to how viral pathogens shape the IgA composition within the gastrointestinal tract.

The persistence of rotavirus-specific IgA in humans and experimental animal models is intriguing and provides the perfect backdrop in which to study the maintenance of viral-specific IgA antibody secreting cells. Plasma cells develop following reactivation of quiescent memory B cells. Antibody secreting cells were thought to be short lived (~2–3 weeks) (221) but this is still being debated. Recently, populations of long-lived plasma cells that produce antibodies for several to many years have been shown to reside in “niches” in the spleen, lymph nodes, and bone marrow (222). The repertoire of these plasma cell niches could be rapidly recalled after temporary depletion indicating the likelihood that these plasma cell niches have a memory component (215). Several characteristics of rotavirus infection in mice indicate that long-lived IgA^+^ plasma cells are generated following rotavirus infection and mediate protective immunity. First, infection of naïve mice results in intestinal and fecal rotavirus-specific IgA that stabilizes around 3 weeks after infection and stays constant over the lifetime of the mouse (132, 151). Second, murine rotavirus infection in mice induces lifetime protection against reinfection (132, 151). Upon re-exposure to rotavirus, viral proteins are not detected in the intestinal tract or in the feces (132, 151) and there is no increase in the titers of rotavirus-specific IgA in the feces (132, 151). The lack of discernible induction of antibody titers following re-exposure suggest: (i) that there is no reactivation of rotavirus-specific memory B cells or generation of new antibody secreting cells and (ii) that the antibody produced by the long-lived plasma cells is sufficient to neutralize the viral challenge. In addition, there is no indication that rotavirus replicates or that antigens are maintained chronically in the mouse past the time when it is detectable in stool (220, 223). This suggests that the plasma cells that produce rotavirus-specific antibody do so in the absence of virus. Work in the gnotobiotic piglet indicates that the bone marrow does not house the IgA response to rotavirus but rather the plasma cells are located in the intestinal lamina propria (200). It remains to be determined whether the specific environment of the intestinal lamina propria facilitates the development and maintenance of long-lived IgA secreting plasma cells that have a memory component (Figure 1).

## Summary

Although IgA is produced in large quantities in the gastrointestinal tract, its importance in the immune response to gastrointestinal viral infections is unclear. In part, this is due to the lack of good animal models in which the pathogenesis, disease, and immune response induced during gastrointestinal viral infection reflects that which occurs in humans. The animal models of rotavirus infection closely mimic many parameters of infection in humans including a profound induction of rotavirus-specific intestinal IgA that correlates with clearance of infection and protection from reinfection. These animal models a currently being used to investigate and characterize the molecular pathways through which the virus induces the intestinal IgA response and will contribute significantly to our understanding of the important role IgA plays in the defense against intestinal virus infections. The models, combined with new technologies, are positioned to reveal new and exciting information as to the location, diversity, and maintenance of long-lived IgA^+^ plasma secreting cells. Furthermore, these studies will facilitate the design and development of future oral vaccines by providing new and more efficient targets to induce protective IgA.

## Conflict of Interest Statement

The authors declare that the research was conducted in the absence of any commercial or financial relationships that could be construed as a potential conflict of interest.
